# Investigation of Macular Choroidal Thickness and Blood Flow Change by Optical Coherence Tomography Angiography After Posterior Scleral Reinforcement

**DOI:** 10.3389/fmed.2021.658259

**Published:** 2021-04-29

**Authors:** Zheng Zhang, Yue Qi, Wenbin Wei, Zi-Bing Jin, Wen Wang, Anli Duan, Wu Liu

**Affiliations:** ^1^Beijing Tongren Eye Center, Beijing Tongren Hospital, Capital Medical University, Beijing Ophthalmology and Visual Sciences Key Laboratory, Beijing, China; ^2^Beijing Institute of Ophthalmology, Beijing Tongren Hospital, Capital Medical University, Beijing, China

**Keywords:** choroidal thickness, choroidal blood flow, swept-source optical coherence tomography angiography, posterior scleral reinforcement, myopic macular degeneration

## Abstract

**Purpose:** This work aimed to study the effect of posterior scleral reinforcement (PSR) on choroidal thickness (CT) and blood flow.

**Methods:** This study included 25 eyes of 24 patients with high myopia ( ≤ -6.0 dioptres or axial length ≥ 26.0 mm) who underwent PSR surgery. All patients completed the 1-month follow-up visit. Myopic macular degeneration (MMD) was graded according to the International Meta-Analysis for Pathologic Myopia (META-PM) classification based on color fundus photographs. Swept-source optical coherence tomography angiography (SSOCTA) was performed to investigate CT, choroidal perfusion area (CPA), and choriocapillaris perfusion area (CCPA) change following PSR surgery.

**Results:** The distribution of MMD categories was 9 (36.0%) in category 1, 10 (40.0%) in category 2, and 6 (24.0%) in category 3 or 4. MMD severity was strongly correlated with CT (all *P* < 0.01) and CPA (all *P* < 0.04). Postoperative CT at each sector increased significantly at 1 week's follow-up, compared to preoperative measures (all *P* < 0.05). Postoperative CPA at subfoveal, superior, inferior, and nasal sectors also increased significantly 1 week after PSR surgery (all *P* < 0.05). Moreover, the increased CT, CPA, and CCPA remain after PSR surgery at 1 month's follow-up, but the difference was not statistically significant.

**Conclusions:** We demonstrated that the CT and choroidal blood flow increased significantly in patients with high myopia who underwent PSR surgery in a short period of time. In addition, the CT and CPA were independently associated with MMD. However, whether the transient improvement of the choroidal circulation could prevent long-term progression of high myopia warrants further study in the future.

## Introduction

The rapidly increasing prevalence of myopia poses one of the most serious public health issues, especially in Asia where pathological myopia has been reported as the primary cause of blindness or low vision in 12–27% of the populations (1–3). The major alterations in pathologic myopia include excessive axial elongation of the globe and associated local ectasia of the posterior sclera, which eventually leads to characterized retinal and choroidal lesions as well as impacts on macular function (4, 5).

Laid between the retina and the sclera in the posterior eye, the highly vascular choroid is essential for maintaining the normal physiology of the eye, such as supplying oxygen and nutrients for the outer retina. There is also substantial evidence that the choroid plays an important role in controlling ocular elongation and refractive error development (6, 7). Growing evidence in the literature has demonstrated that a short-term thickening of the choroid leads to a prolonged decrease in extracellular matrix molecule synthesis and a slowing of eye growth (8–10). Therefore, controlling choroidal thinning could be a crucial approach to maintain emmetropia and reduce the incidence of severe myopic maculopathy.

As a treatment targeting the posterior pole of the eye, posterior scleral reinforcement (PSR) was first proposed by Shevelev in 1930 and later modified by Snyder and Thompson in 1972 (11). PSR has been considered an effective and safe surgical method for stabilization of the axial elongation and prevention of high myopic complications (5, 12). The mechanism by which PSR may slow down the elongation of the eyeball was presumably due to the direct mechanical force of the reinforcement band and the scleral remodeling and improvement of microcirculation within the macula (13, 14). However, whether or not PSR could change the choroidal thickness (CT) and choroidal blood flow is still under debate due to lack of satisfactory quantitative methods (5, 15).

Swept-source optical coherence tomography angiography (SSOCTA) has been introduced as a new non-invasive, quantitative approach to visualize and evaluate the choroidal microvasculature (16). In comparison with enhanced depth imaging (EDI) by spectral domain optical coherence tomography (SDOCT), swept-source optical coherence tomography (SSOCT) with a light source of 1,050 nm can provide a better-visualized full-thickness choroid and enable more accurate measurements of the choroidal structure (17).

The purpose of our study was to observe the changes of CT and the choroidal vasculature structure, as well as axial length (AL), best corrected visual acuity (BCVA), and spherical equivalent (SE), after PSR in patients with pathological myopia.

## Methods

### Highly Myopic Patients Received PSR Surgery

This was a prospective, observational clinical study of consecutive patients with high myopia aged 31–68 years undergoing PSR surgery. Twenty-four patients (25 eyes) diagnosed with pathological myopia were recruited from the Beijing Tongren Eye Center from October 2019 to October 2020. The inclusion criteria were as follows: manifest SE of ≤ -6.0 dioptres (D) with increases ≥-1.00 D/year, AL of ≥26.0 mm with annual progressive growth of >0.5 mm for 2 years or more, and posterior staphyloma confirmed by B-ultrasound scan (IOL Master, Carl Zeiss Inc., Jena, Germany). Exclusion criteria included the following: (1) any ocular disease that may affect measurements of the choroid, such as corneal opacities, dense cataract, central serous chorioretinopathy, polypoidal choroidal vasculopathy, choroidal neovascularization, and non-myopia-related macular scarring; (2) history of intraocular surgery and previous retinal photocoagulation or photodynamic therapy; (3) presence of other ocular diseases such as glaucoma, tumor, uveitis, and retinal vascular disease. This study was approved by the Institutional Ethics Committee of Beijing Tongren Hospital, Capital Medical University, and conducted in accordance with the tenets of the Declaration of Helsinki. All participants provided signed informed consent for their participation.

### Clinical Examination

Patients were followed up at 1 week and 1 month after PSR surgery. Preoperative and postoperative examinations included logMAR BCVA, slit lamp biomicroscopic examination, AL using IOL Master (Carl Zeiss Inc., Jena, Germany), pupil-dilated funduscopy, color fundus photography (Hybrid Digital Mydriatic Retinal Camera CX-1, Canon Inc., Tokyo, Japan), and manifest refraction performed by qualified optometrists. SE was calculated using the spherical power plus half of the cylindrical power.

### Grading of MMD

**Figure 1:**
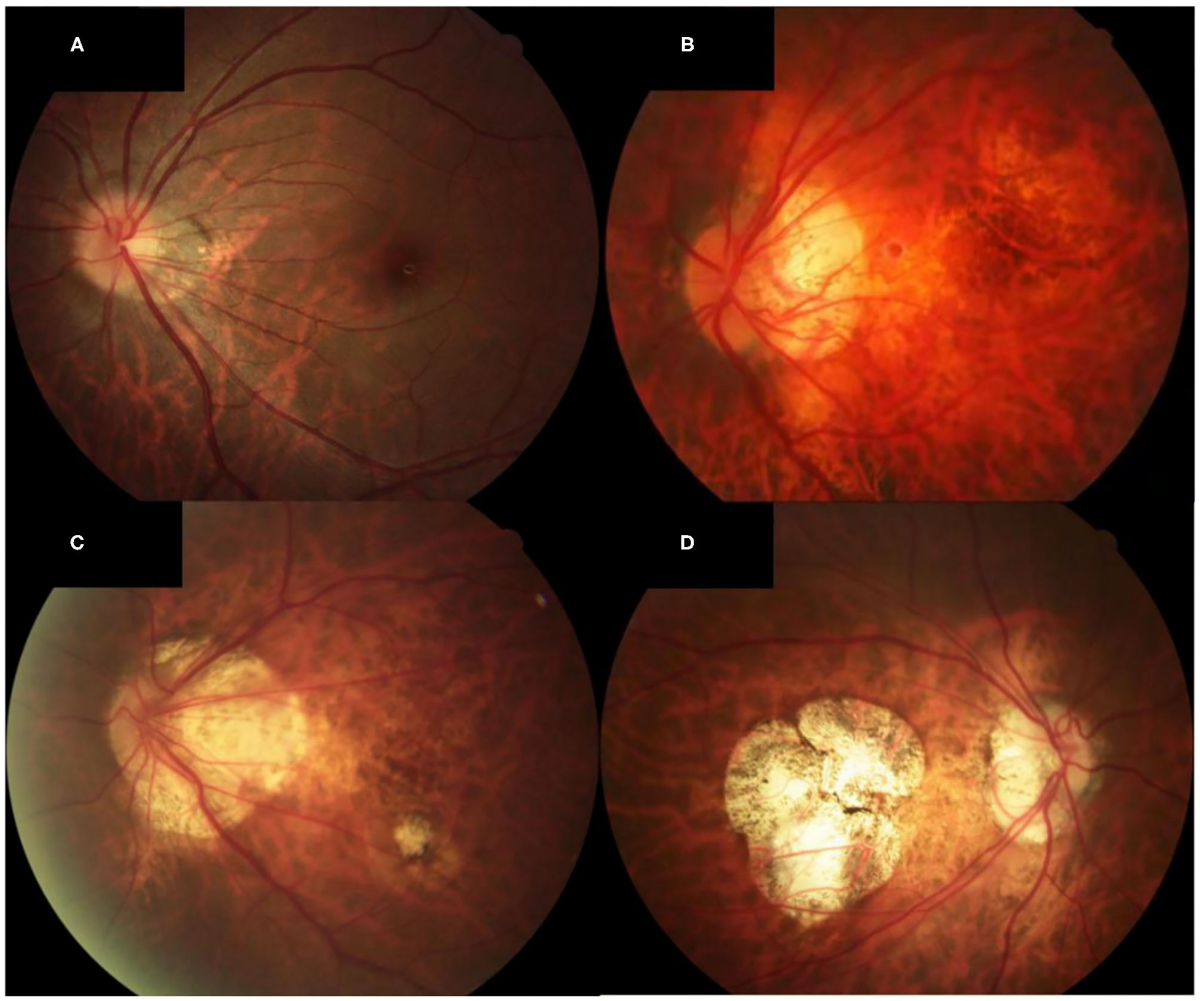
Fundus photographs of eyes showing different MMD severity grades. **(A)** Fundus photograph of the left eye of a patient with META-PM category 1 (AL of 25.64, SE of −8.4 D, and BCVA of 0 logMAR units). Only the tessellated fundus can be seen on the fundus photograph. **(B)** Fundus photograph of the left eye of a patient with META-PM category 2 (AL of 29.36, SE of −16.5 D, and BCVA of 0.2 logMAR units). Diffuse chorioretinal atrophy is seen on the fundus photograph. **(C)** Fundus photograph of the left eye of a patient with META-PM category 3 (AL of 28.98, SE of −16.0 D, and BCVA of 0.4 logMAR units). Both diffuse and patchy chorioretinal atrophy can be seen on the fundus photograph. **(D)** Fundus photograph of the right eye of a patient with META-PM category 4 (AL of 31.04, SE of −20.0 D, and BCVA of 1.3 logMAR units). Macular atrophy is seen on the fundus photograph.

According to the international META-PM classification (18), the MMD severity was defined and classified into the following categories: no macular lesions was defined as META-PM category 0; tessellated fundus was defined only as META-PM category 1; diffuse chorioretinal atrophy was defined as META-PM category 2; patchy chorioretinal atrophy was defined as META-PM category 3; and macular atrophy was defined as META-PM category 4 (Figure 1). Two ophthalmologists, masked to patient characteristics, performed the grading of MMD severity. Discrepancies were adjudicated by a senior fundus disease specialist.

### Measurement of CT, Choroidal Perfusion Area, and Choriocapillaris Perfusion Area

**Figure 2:**
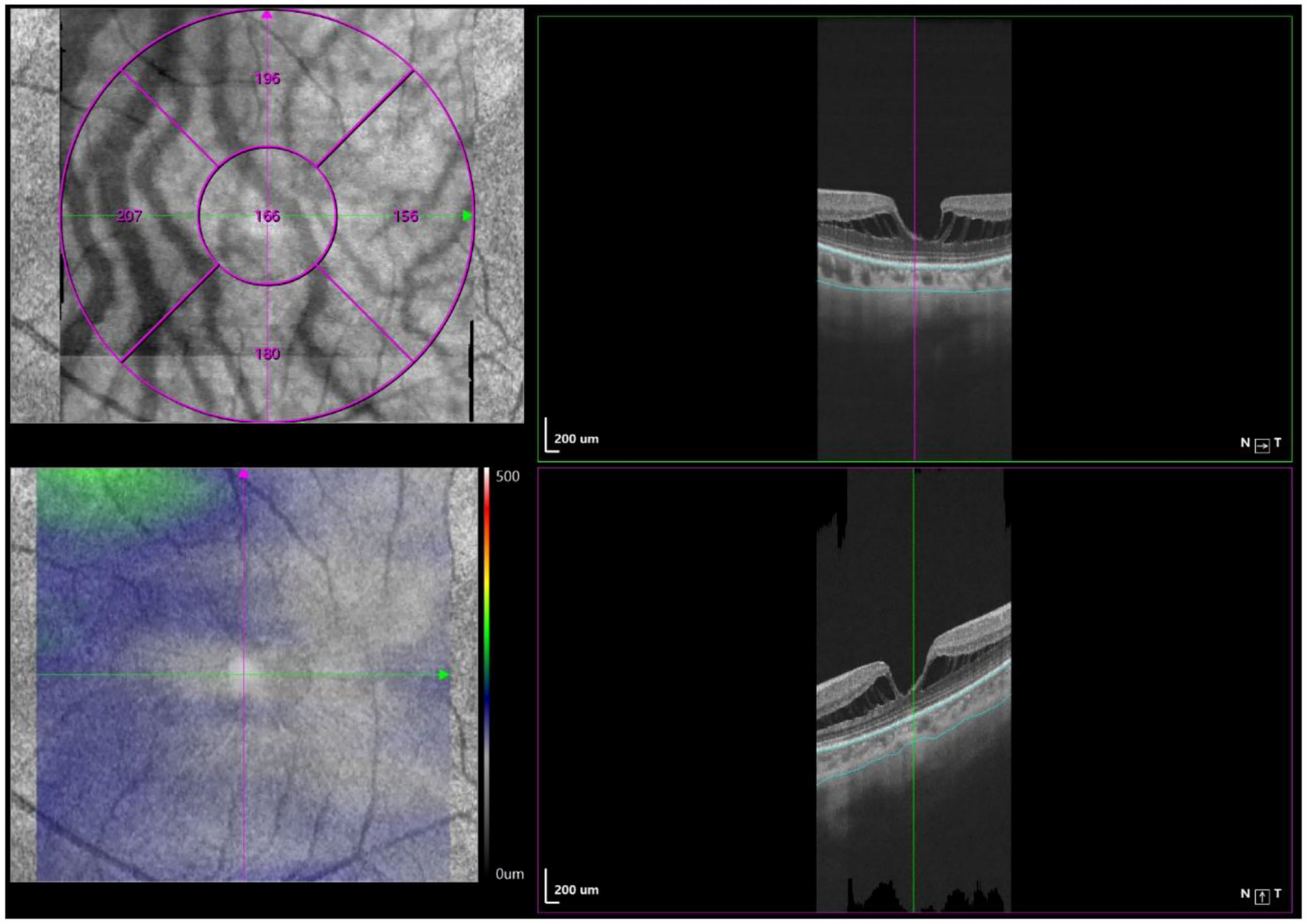
Macular CT measurement by swept-source OCT angiography. Macular CT was separately calculated in 5 regions (fovea, tempo, superior, nasal, and inferior) based on ETDRS contour. The choroid was defined as the volume from the basal border of the RPE–Bruch's membrane complex to the chorioscleral junction.

**Figure 3:**
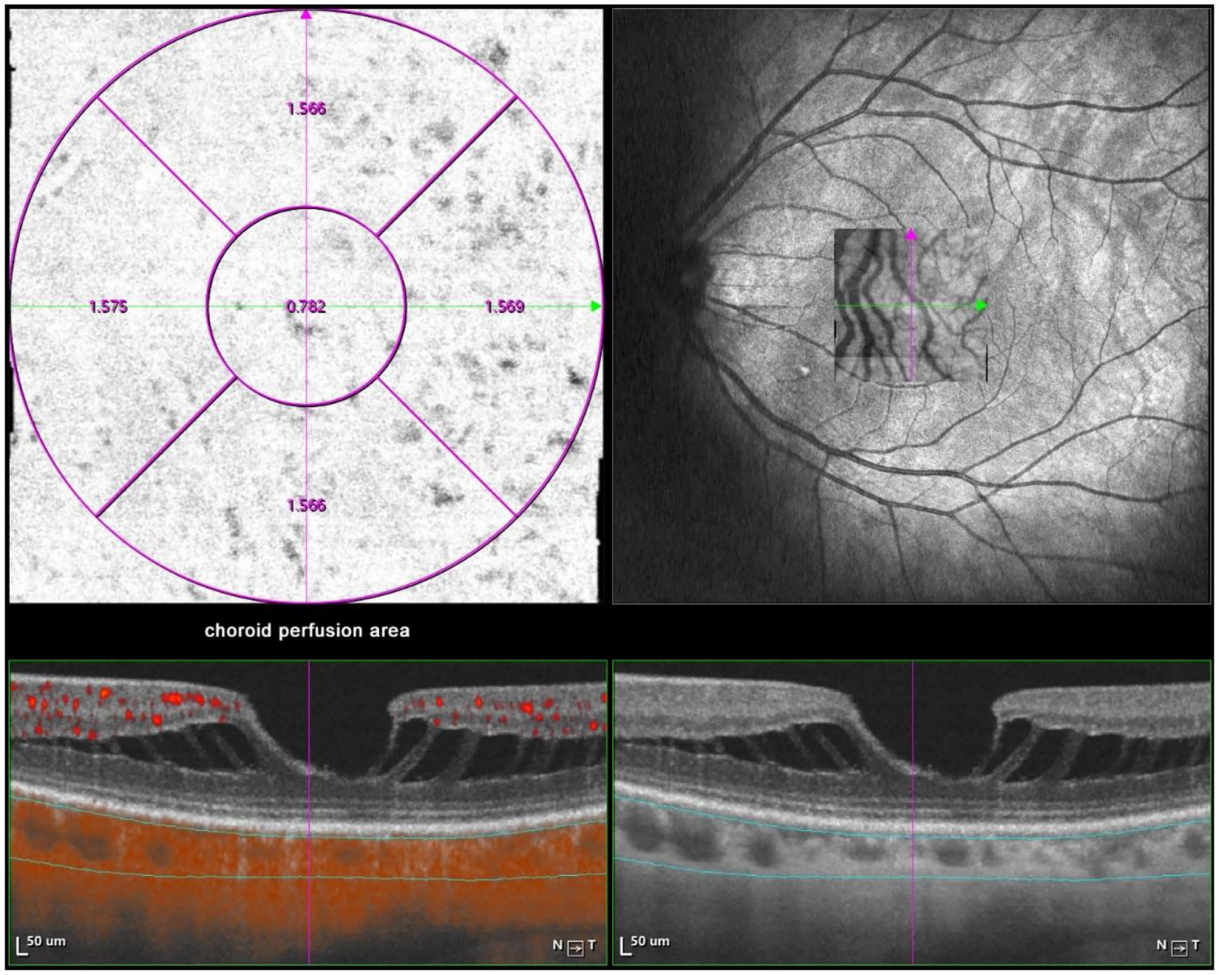
Macular CPA measurement by SSOCTA. Macular CPA was separately calculated in five regions (foveal, temporal, superior, nasal, and inferior) based on the ETDRS contour. The CPA was defined as the area of blood flow to the whole *en face* scanning area at the choroidal layer on OCTA images.

**Figure 4:**
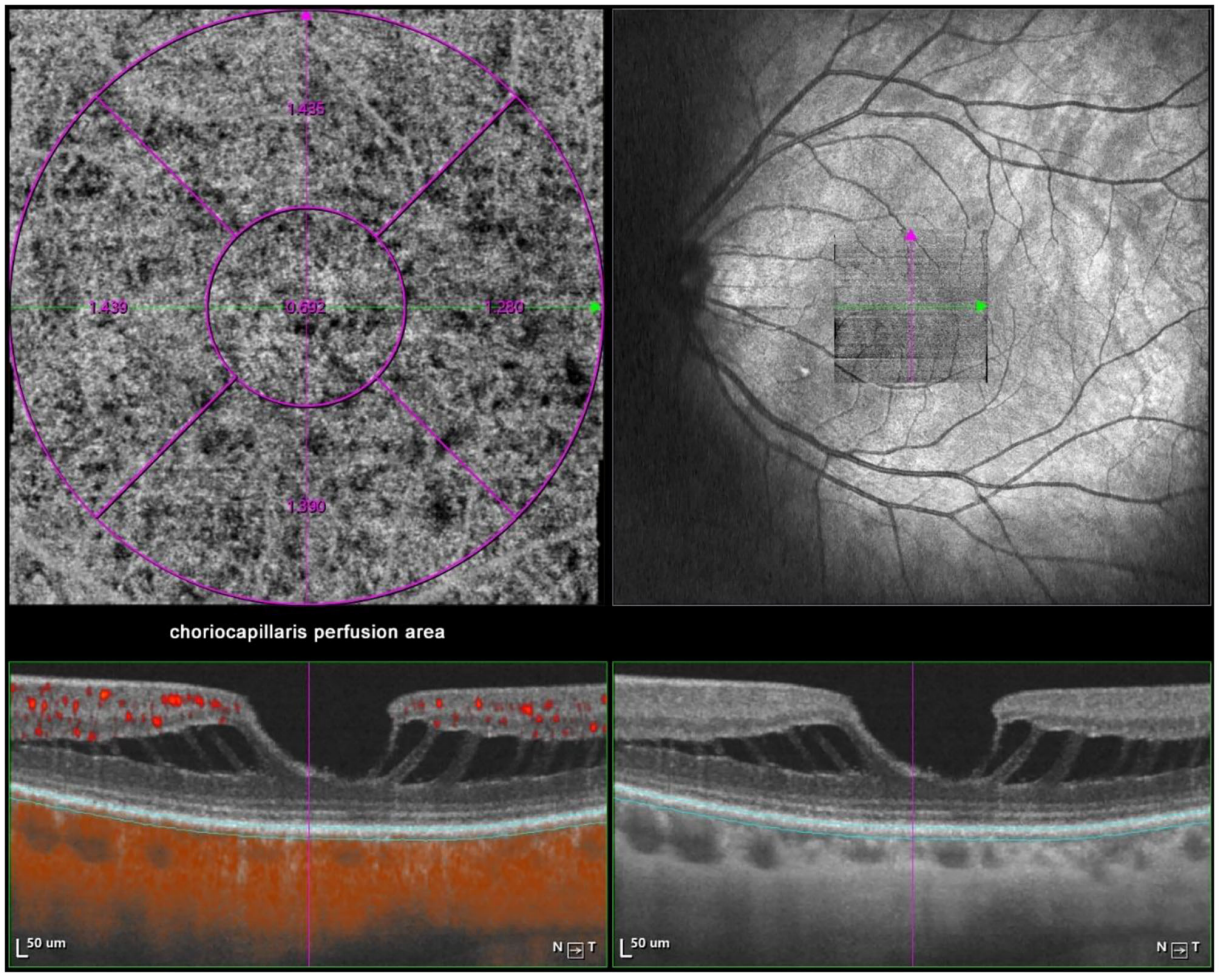
Macular CCPA measurement by SSOCTA. Macular CCPA was separately calculated in five regions (foveal, temporal, superior, nasal, and inferior) based on the ETDRS contour. The CCPA was defined as the area of blood flow to the whole *en face* scanning area at the choriocapillaris layer on OCTA images.

Optical coherence tomography angiography (OCTA) scans were obtained using the commercial VG200 SSOCTA device with a light source of 1,050 nm. Detailed information on the acquisition protocols for this device has been previously reported (16). Both optical coherence tomography (OCT) and OCTA data were obtained with a raster scan protocol of 512 × 512 B-scans, which covered an area of 3 × 3 mm centered on the fovea. The macular region was divided into foveal, temporal, superior, nasal, and inferior sectors based on the Early Treatment Diabetic Retinopathy Study (ETDRS) contour. The choroid in OCT was defined as the volume starting at the retinal pigment epithelium (RPE)–Bruch's membrane complex and ending at the chorioscleral junction (Figure 2). The choriocapillaris was defined as the volume from the basal border of the RPE–Bruch's membrane complex to approximately 20 μm beneath the RPE–Bruch's membrane complex. The CPA/CCPA was defined as the area occupied by blood vessels in a 2D retina projection image. The projection image was acquired by projecting a 3D angiography volume data of the choroidal/choriocapillaris layer onto a 2D imaging plane, which can also be called as an *en face* image. The presence of perfusion was directly indicated by an angiography signal. An algorithm was designed to separate the foreground (blood vessel) pixels from background (non-vessel tissue) pixels, by properly segmenting the image from the perspective of angiography signal strength. The perfusion area was calculated as the sum of the area of all pixels that exceed the threshold (Figures 3, 4). The magnification for imaging the fundus using OCT is different in the myopic eye due to the elongation of the eye. Hence, in the present study, Bennett's formula was used to determine a scaling factor of the OCT angiograms for adjustment of the ocular magnification [scaling factor = 3.382 × 0.013062 × (AL – 1.82)] (19).

### Surgical Procedure

**Figure 5:**
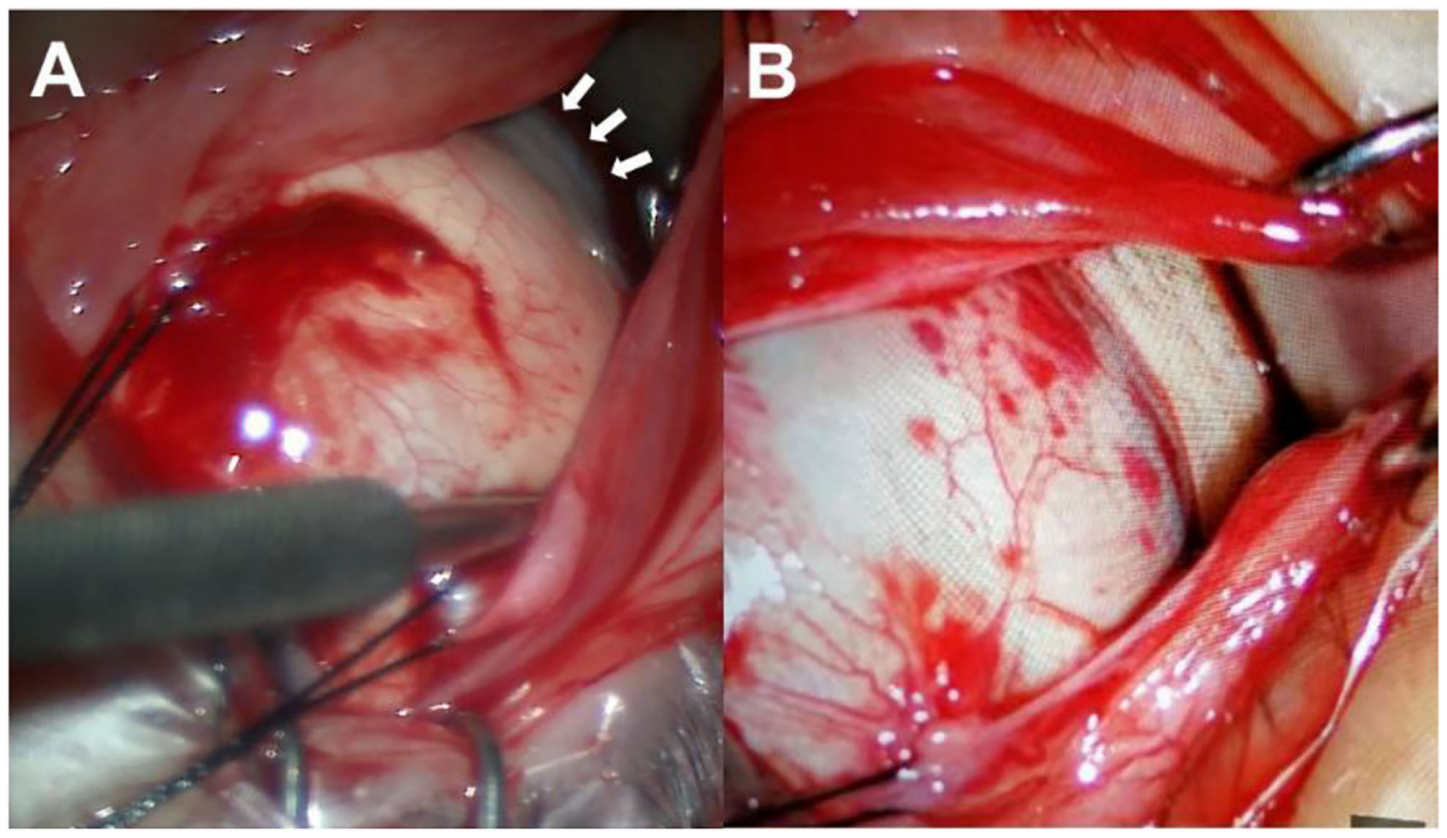
Surgical procedures. **(A)** The inferior and lateral rectus muscles were maneuvered by traction sutures while the eyeball was pulled toward the superior nasal side to expose the scleral staphyloma (white arrows). **(B)** The scleral strip was inserted underneath the lateral rectus, inferior oblique, and inferior rectus muscles and wrapped around the posterior pole and scleral staphyloma.

The surgical techniques of PSR were basically following the modified Snyder–Thompson procedure. All PSR procedures were performed by the same surgeon (Yue Qi). Under general anesthesia, a 210° peritotomy of the conjunctiva was performed along the inferior–temporal axis of the limbus, and the inferior and lateral rectus muscles were isolated and exposed. The two muscles were maneuvered by traction sutures while the eyeball was pulled toward the superior nasal side. After the inferior oblique muscle was isolated, a homologous human scleral strip with a width of 6–10 mm was sequentially inserted underneath the lateral rectus, inferior oblique, and inferior rectus muscles. The superior end of the strip was fixed at the nasal side of the scleral insertion of the superior rectus muscle, while the inferior end of the strip was anchored at the nasal side of the scleral insertion of the inferior rectus muscle. The scleral strip was stretched into a U-shape to wrap around the posterior pole and scleral staphyloma corresponding to the macular area and was flattened with the help of strabismus hooks. The relative position between the scleral strip and optic nerve was checked with a strabismus hook. The distance was kept at approximately 1 mm to ensure that the strip covered the foveal region without compressing the optic nerve (20) (Figure 5).

### Statistical Analysis

Statistical analysis was performed using SPSS software (version 25.0; IBM, Chicago, USA). Descriptions of the quantitative data were presented as the mean ± standard deviation (SD). The independent-samples *t*-test was used for comparing continuous data between two groups. Repeated-measures analysis of variance (RMANOVA) with the *post-hoc* least significant difference (LSD) test was used to assess the differences among the eyes before PSR surgery, 1 week after PSR surgery, and 1 month after PSR surgery. Spearman correlation coefficients were calculated to analyze the correlation between AL, SE, and BCVA with MMD severity. *P*-values < 0.05 were considered significant. The software used for data visualization is RStudio.

## Results

### Demographic and Clinical Data

Twenty-four patients with 25 eyes diagnosed with pathological myopia were recruited and completed 1 week and 1 month postoperative follow-ups. Table 1 shows the baseline characteristics of these eyes. The mean age was 50.5 ± 11.5 years, the mean AL was 29.23 ± 1.87 mm, the mean SE was −15.3 ± 4.3 D, and the mean BCVA was 0.40 ± 0.25. Nine (36.0%) eyes had tessellated fundus only (category 1), 10 (40.0%) eyes had diffuse chorioretinal atrophy (category 2), and six (24.0%) eyes had patchy chorioretinal atrophy (category 3) or macular atrophy (category 4). MMD severity was significantly correlated with AL (correlation coefficient |*r*| = 0.72, *P* < 0.001), SE (*r* = −0.87, *P* < 0.001), and BCVA (*r* = 0.49, *P* = 0.02). Patients with severe MMD had longer AL and more myopic refractive error compared with eyes with mild MMD (Table 1). The mean SE post PSR was −13.5 ± 5.9 D. The mean BCVA post PSR was 0.44 ± 0.23. No significant differences were found before and after PSR (all *P* > 0.16).

**Table 1:** Baseline characteristics of study eyes.

	**Number of subjects**			
**Baseline characteristic**	**All** ** (*n* = 24)**	**META-PM category 1** ** (*n* = 9)**	**META-PM category 2** ** (*n* = 9)**	**META-PM categories** ** 3 and 4 (*n* = 6)**
Mean age, years	50.5 ± 11.5	49.1 ± 13.4	47.7 ± 10.0	56.7 ± 10.0
Male, %	8 (33.3)	2 (22.2)	4 (44.4)	2 (33.3)
	**Number of eyes**			
**Baseline characteristic**	**All** ** (*****n*** **=** **25)**	**META-PM category 1** ** (*****n*** **=** **9)**	**META-PM category 2** ** (*****n*** **=** **10)**	**META-PM categories** ** 3 and 4 (*****n*** **=** **6)**
AL, mm	29.23 ± 1.87	27.60 ± 0.90	29.80 ± 1.07^†^	30.75 ± 2.26^†^
SE, D	−15.3 ± 4.3	−10.77 ± 1.96	−16.50 ± 2.79^†^	−20.00 ± 2.15^†^^‡^
logMAR BCVA	0.40 ± 0.25	0.30 ± 0.35	0.40 ± 0.24	0.48 ± 0.12

† *P < 0.05 by independent-samples t-test vs. META-PM category 1*.

‡ *P < 0.05 by independent-samples t-test vs. META-PM category 2*.

### CT, CPA, and CCPA in Eyes With Different MMD Grades

**Figure 6:**
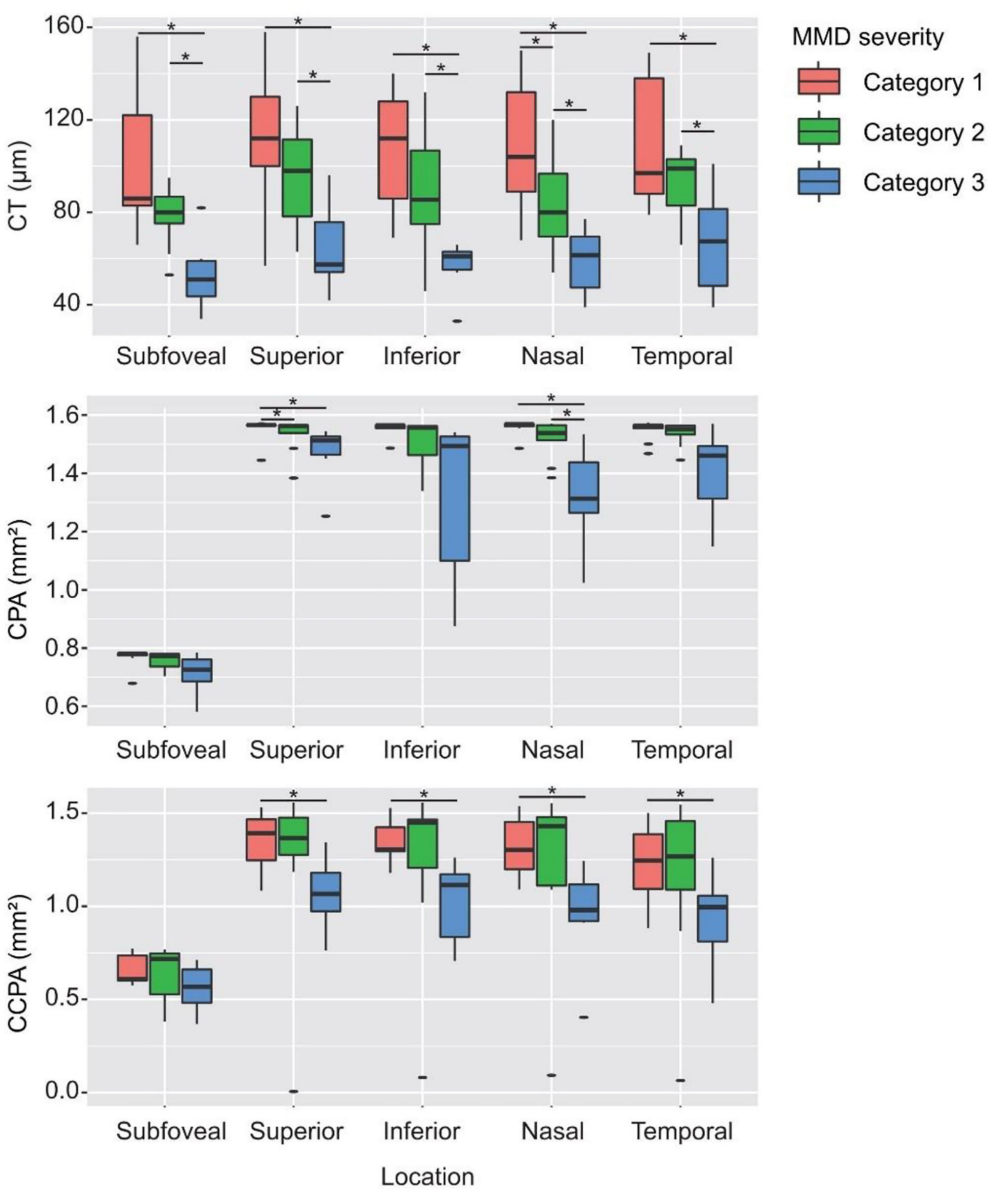
CT, CPA, and CCPA in eyes with different MMD severity grades. *Statistically significant at *P* < 0.05 when the META-PM category 1 group or the META-PM category 2 group was compared with the META-PM category 3 group and when the META-PM category 1 group was compared with the META-PM category 2 group.

Table 2 shows the CT, CPA, and CCPA in eyes with different MMD grades. The subfoveal, superior, inferior, nasal, and temporal CTs were significantly thinner in eyes with META-PM category 3 or 4 at baseline than in eyes with META-PM category 1 (all *P* < 0.009) and in eyes with META-PM category 2 (all *P* < 0.027). Nasal CT was significantly thinner in eyes with META-PM category 2 than in eyes with META-PM category 1 (83.10 ± 20.65 vs. 107.78 ± 29.48 μm, *P* = 0.048). The subfoveal, superior, inferior, and temporal CTs were thinner in eyes with META-PM category 2 than in eyes with META-PM category 1, but the difference was not statistically significant (Figure 6 and Table 2). We further evaluated the correlation between MMD severity and CT (Table 3). CT was strongly correlated with MMD severity based on META-PM classification (all *P* < 0.01). In addition, subfoveal CT had a strong correlation with BCVA (*r* = −0.49, *P* = 0.021).

**Table 2:** CT, CPA, and CCPA in eyes with different MMD severity grades.

**CT/CPA/CCPA**	**META-PM category 1** ** (*n* = 9)**	**META-PM category 2** ** (*n* = 10)**	**META-PM categories 3 and 4 (*n* = 6)**
**CT**, **μm**			
Subfoveal	101.89 ± 32.18	78.20 ± 12.73	53.50 ± 16.78^†^^‡^
Superior	113.44 ± 30.53	96.10 ± 21.39	64.67 ± 19.96^†^^‡^
Inferior	106.56 ± 25.61	90.40 ± 26.93	56.33 ± 12.16^†^^‡^
Nasal	107.78 ± 29.48	83.10 ± 20.65^†^	59.00 ± 15.09^†^^‡^
Temporal	111.11 ± 28.69	92.30 ± 15.53	67.17 ± 24.19^†^^‡^
**CPA, mm**^**2**^			
Subfoveal	0.77 ± 0.03	0.76 ± 0.03	0.71 ± 0.07
Superior	1.55 ± 0.04	1.53 ± 0.06^†^	1.47 ± 0.11^†^
Inferior	1.55 ± 0.03	1.51 ± 0.08	1.32 ± 0.31
Nasal	1.56 ± 0.03	1.52 ± 0.07	1.32 ± 0.18^†^^‡^
Temporal	1.55 ± 0.04	1.54 ± 0.04	1.40 ± 0.16
**CCPA, mm**^**2**^			
Subfoveal	0.66 ± 0.08	0.64 ± 0.14	0.56 ± 0.13
Superior	1.36 ± 0.16	1.24 ± 0.48	1.07 ± 0.20^†^
Inferior	1.34 ± 0.12	1.23 ± 0.46	1.02 ± 0.23^†^
Nasal	1.33 ± 0.16	1.21 ± 0.46	0.95 ± 0.29^†^
Temporal	1.23 ± 0.21	1.15 ± 0.47	0.93 ± 0.27^†^

† *P < 0.05 by independent-samples t-test vs. META-PM category 1*.

‡ *P < 0.05 by independent-samples t-test vs. META-PM category 2*.

**Table 3:** Correlation between CT, CPA, and CCPA with MMD severity, AL, SE, and BCVA.

**CT/ST**	**MMD severity**		**AL**		**SE**		**BCVA**	
	***r***	***P*-value**	***r***	***P*-value**	***r***	***P*-value**	***r***	***P*-value**
**CT**, **μm**								
Subfoveal	−0.66	<0.001^†^	−0.37	0.07	0.35	0.10	−0.49	0.021^†^
Superior	−0.62	0.001^†^	−0.46	0.02^†^	0.39	0.07	−0.21	0.35
Inferior	−0.67	<0.001^†^	−0.55	0.01^†^	0.62	0.002^†^	−0.26	0.25
Nasal	−0.67	<0.001^†^	−0.53	0.01^†^	0.54	0.01^†^	−0.19	0.40
Temporal	−0.49	0.01^†^	−0.41	0.04^†^	0.36	0.09	−0.21	0.35
**CPA, mm**^**2**^								
Subfoveal	−0.41	0.04^†^	−0.26	0.20	0.04	0.86	−0.34	0.12
Superior	−0.65	<0.001^†^	−0.49	0.01^†^	0.32	0.14	−0.40	0.07
Inferior	−0.64	0.001^†^	−0.63	0.001^†^	0.51	0.01^†^	−0.46	0.03^†^
Nasal	−0.71	<0.001^†^	−0.62	0.001^†^	0.43	0.04^†^	−0.42	0.05
Temporal	−0.48	0.02^†^	−0.41	0.04^†^	0.26	0.24	−0.19	0.39
**CCPA, mm**^**2**^								
Subfoveal	−0.27	0.20	−0.18	0.41	0.01	0.97	−0.47	0.03^†^
Superior	−0.41	0.04^†^	−0.29	0.17	0.09	0.68	−0.54	0.01^†^
Inferior	−0.44	0.03^†^	−0.26	0.22	0.21	0.34	−0.50	0.02^†^
Nasal	−0.47	0.02^†^	−0.28	0.19	0.14	0.53	−0.65	0.001^†^
Temporal	−0.32	0.13	−0.23	0.29	0.06	0.78	−0.43	0.05

† *P < 0.05 by Spearman correlation coefficients*.

Superior and nasal CPAs were significantly lower in eyes with META-PM category 3 or 4 at baseline than in eyes with META-PM category 1 (all *P* < 0.05). The subfoveal, inferior, and temporal CPAs were lower in eyes with META-PM category 3 or 4, but the difference was not statistically significant. All superior, inferior, nasal, and temporal CCPAs were significantly lower in eyes with META-PM category 3 or 4 at baseline than in eyes with META-PM category 1 (all *P* < 0.031). Subfoveal CCPA was lower in eyes with META-PM category 3 or 4, but the difference was not statistically significant (Figure 6 and Table 2). CPA was correlated with MMD severity (all *P* < 0.04). Superior, inferior, and nasal CCPAs were correlated with MMD, but the correlation was weaker than that between CPA and MMD. In addition, CCPA had a moderate correlation with BCVA (all *P* < 0.05) (Table 3).

### Postoperative Outcomes of CT, CPA, and CCPA

**Figure 7:**
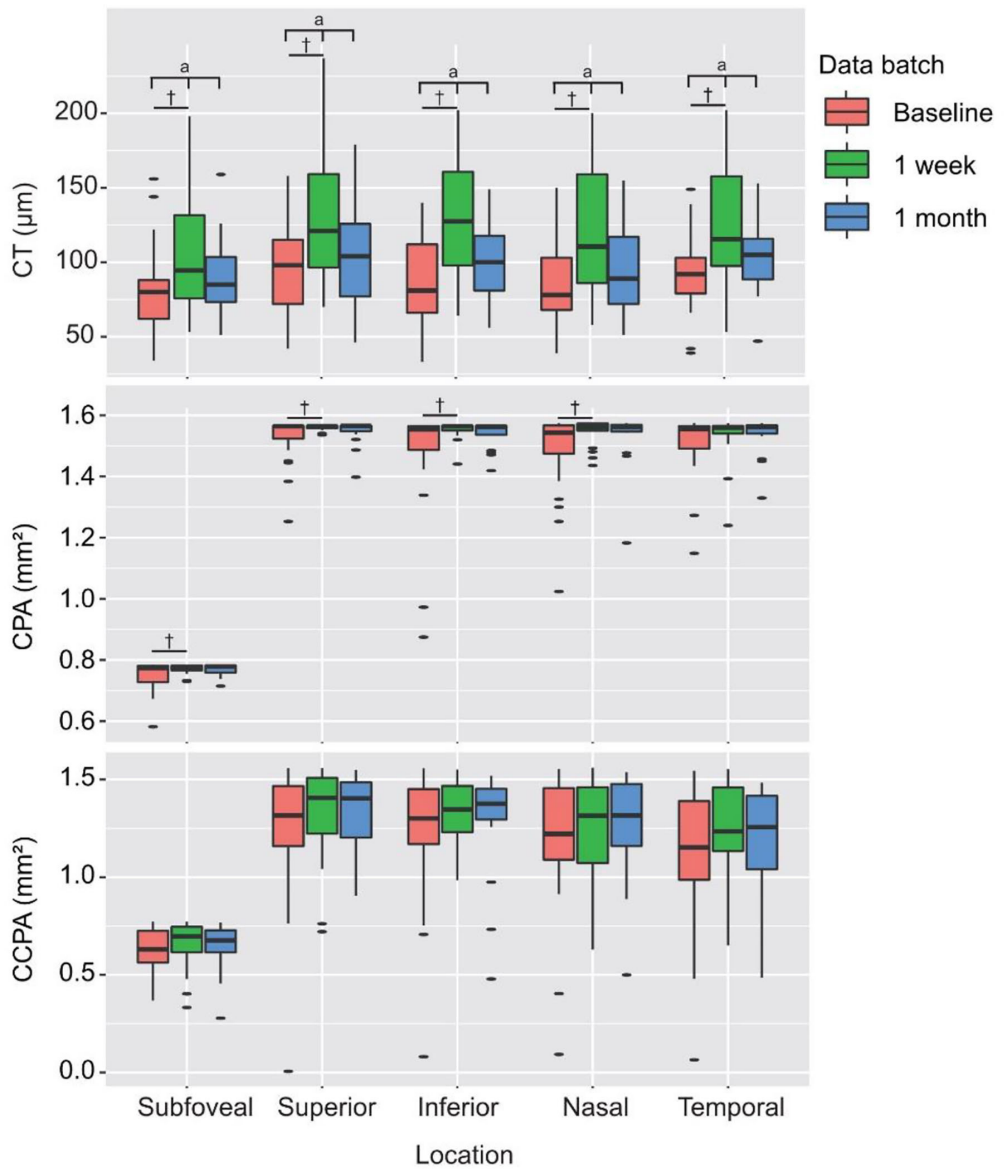
Preoperative and postoperative measurements of CT, CPA, and CCPA of the center subfield and parafovea (superior, inferior, nasal, and temporal subfields). ^a^Statistically significant at *P* < 0.05 by RMANOVA, ^†^Statistically significant at *P* < 0.05 by multiple comparisons (LSD *post-hoc* test) vs. the baseline.

Postoperative outcomes of CT, CPA, and CCPA are presented in Table 4. The CT of the center subfield and parafoveal subfields increased significantly after PSR surgery at 1 week's follow-up (all *P* < 0.01). The subfoveal, superior, inferior, and nasal CPAs increased significantly after PSR surgery at 1 week's follow-up (all *P* < 0.05). The temporal CPAs were increased after PSR surgery at 1 week's follow-up, but the difference was not statistically significant. CCPA did not change significantly compared to preoperation measures. The increased CT, CPA, and CCPA remain after PSR surgery at 1 month's follow-up, but the difference was not statistically significant (all *P* > 0.05) (Figure 7 and Table 4).

**Table 4:** Preoperative and postoperative measurements (mean ± SD) of CT, CPA, and CCPA of the center subfield and parafovea (superior, inferior, nasal, and temporal subfields).

	**Baseline**	**1 week**	**1 month**	***P*-value^***a***^**
CT subfoveal (μm)	80.80 ± 28.65	107.35 ± 42.53^†^	89.56 ± 26.43	0.033
CT superior (μm)	94.80 ± 30.38	126.90 ± 45.20^†^	105.78 ± 35.57	0.019
CT inferior (μm)	88.04 ± 30.06	128.95 ± 43.30^†^	99.33 ± 27.21	0.001
CT nasal (μm)	86.20 ± 29.34	118.85 ± 41.64^†^	95.67 ± 30.76	0.008
CT temporal (μm)	93.04 ± 27.87	125.10 ± 42.00^†^	105.39 ± 27.10	0.007
CPA subfoveal (μm)	0.75 ± 0.05	0.77 ± 0.02^†^	0.77 ± 0.02	0.068
CPA superior (μm)	1.52 ± 0.07	1.56 ± 0.01^†^	1.55 ± 0.04	0.080
CPA inferior (μm)	1.48 ± 0.18	1.55 ± 0.03^†^	1.54 ± 0.04	0.087
CPA nasal (μm)	1.48 ± 0.13	1.55 ± 0.04^†^	1.53 ± 0.09	0.108
CPA temporal (μm)	1.51 ± 0.10	1.53 ± 0.08	1.53 ± 0.06	0.534
CCPA subfoveal (μm)	0.63 ± 0.12	0.65 ± 0.13	0.65 ± 0.12	0.771
CCPA superior (μm)	1.24 ± 0.33	1.32 ± 0.25	1.34 ± 0.20	0.466
CCPA inferior (μm)	1.22 ± 0.33	1.33 ± 0.16	1.29 ± 0.28	0.399
CCPA nasal (μm)	1.19 ± 0.35	1.26 ± 0.26	1.27 ± 0.27	0.621
CCPA temporal (μm)	1.12 ± 0.35	1.25 ± 0.26	1.19 ± 0.28	0.420

*CT, choroidal thickness; CPA, choroidal perfusion area; CCPA, choriocapillaris perfusion area*.

a *RMANOVA*.

† *P < 0.05 by multiple comparisons (LSD post-hoc test) vs. the baseline*.

## Discussion

In this study, we demonstrated strong correlations of CT and CPA with MMD severity and significant thinning of the choroid in eyes with severe MMD compared to eyes with mild MMD. Our data support the importance of CT and choroidal blood flow in the pathogenesis of MMD. We also found that there was a transient increase in CT and CPA after PSR surgery at 1 week's follow-up. However, whether this transient improvement of the CT and choroidal blood flow could slow the elongation of the eyeball and prevent high myopic complications still needs to be further studied.

In this study, we used the international META-PM classification to grade MMD severity. Our findings showed that MMD severity was well-correlated with anatomical and functional parameters (AL, SE, and BCVA). However, how eyes with high myopia develop the typical atrophic and degenerative changes seen in MMD, while others do not, is currently unclear. Mechanical stretching of the retina by axial elongation and choroidal ischemia are the most likely mechanisms for MMD (4). Wong et al. measured posterior scleral thickness and CT with SSOCT in 62 eyes with high myopia. A thinner choroid was found to be significantly correlated with MMD severity (21). The strength of the correlation with MMD severity was stronger for CT than it was for scleral thickness, suggesting that a vascular mechanism involving choroidal ischemia may be playing a more prominent role in the pathogenesis of MMD than a mechanical mechanism related to scleral stretch. But CT is a measure of the structure of the choroid. Therefore, it may not truly demonstrate the functional aspect of choroidal blood flow. In our study, we included CPA and CCPA as biomarkers of choroidal vascularity. Our findings showed that CPA was moderately well-correlated with MMD severity. Both CPA and CCPA tend to be lower in eyes with severe MMD compared to eyes with mild MMD, although the difference was not statistically significant in some regions. Previous studies have found that more severe myopia levels were associated with longer axial elongation (22) and that eyes with severe MMD were at higher risk of MMD progression than eyes with mild MMD (23). Considering the relationship between CPA and MMD severity, these results may present a role for choroidal vascularity in the myopia development and progression.

The nutrient supply of the choroid mainly comes from the short-posterior ciliary artery. As one of the most highly vascularized tissues of the body, the choroid accounts for 65% of the blood supply within the eye; hence, it is reasonable to assume that the progression of pathological myopia is linked to the structural and functional alterations of the choroid (5, 24–26). Studies have shown that CT alterations occurred earlier than the fundus changes and that visual function abnormalities occurred in the early stage of pathologic myopia (27, 28). Nickla et al. reported that AL grew faster in eyes with thinner CT than in eyes with thicker choroids (29). Flores-Moreno et al. have reported that CT decreased by 25.9 ± 2.1 μm for each additional millimeter of AL (27). Currently, with the development of OCTA, a few available clinical studies have demonstrated that choroidal vascularity and choriocapillaris blood flow were lower in eyes with greater myopia (30–32). Furthermore, some recent experimental studies may help make a definitive conclusion regarding the relationship between choroidal vascularity and myopia development. Wu et al. (33) demonstrated that scleral hypoxia played an essential role in scleral remodeling during myopia progression. Zhou et al. (34) found that increased choroidal blood perfusion inhibited eyeball elongation and myopia development via attenuating scleral hypoxia in guinea pigs. In this study, we also found that AL is especially associated with parafoveal subfields CT and CPA.

Some studies of high myopia have also found a significant correlation between subfoveal CT and visual acuity, with a thinner choroid being correlated with poorer vision in highly myopic eyes (35–37). In accordance with these researches, in this study, we also found a significant correlation of subfoveal CT with BCVA. Moreover, we found that all center subfield and parafoveal subfield CCPAs were correlated with BCVA. Although the exact mechanism linking a thinner choroid with poor vision is not clearly revealed from the above studies, it has been proposed that the marked thinning of the choroid in eyes with high myopia may affect the function of the photoreceptor, eventually resulting in loss of vision.

PSR is believed to be a safe and reliable method for preventing axial elongation, halting further myopia development, and preserving vision acuity (5, 38). It is reasonable to speculate that PSR surgery could potentially influence the CT and choroidal blood flow as well. In the past years, various techniques had been tried to demonstrate the ocular blood flow in pathologic myopia, such as fundus fluorescein angiography (FFA), indocyanine green angiography (ICGA), color Doppler imaging (CDI). However, they are not satisfactory tools to quantitatively evaluate the microcirculation of the retina and choroid; thus, there are few studies on the microcirculation changes post PSR surgery in highly myopic eyes by far. For the past few years, OCTA was proven to be feasible in detecting choroidal and retinal microvasculature non-invasively and quantitatively. However, in a previous study, Mo et al. did not find a significant change of choriocapillary flow density after PSR, compared to untreated highly myopic eyes with matched AL and SE by RTVue XR OCT with the Angio Retina mode (20). Zhang et al. reported that choriocapillary flow density and CT did not change significantly after PSR surgery either (39). Regarding the disappointing results in previous researches, traditional SDOCT angiography (SDOCTA) may be also limited in detecting changes in choroidal blood flow.

In our present study, we evaluated the CT and choroidal vascularity by VG200 since VG200 is an SSOCTA with more accurate images for choroidal vascularity evaluation. We found that CT and subfoveal, superior, inferior, and nasal CPAs increased significantly after PSR surgery at 1 week's follow-up. But no significant differences were found at 1 month's follow-up. Therefore, it is possible that PSR can lead to a short-term increase of choroidal blood flow but cannot maintain the blood flow in pathologic myopic eyes for a long time. This is probably due to the separation of the sclera between the posterior choroid and the reinforcement band; thus, the secondary non-specific inflammatory reaction could only mildly improve choroidal blood flow. But by the evidence that PSR could prevent eye elongation and halt thinning and atrophy of the choroid (5), there is reason to believe that deterioration of circulation was prevented. Aside from the effects of PSR surgery upon the choroid, a range of other factors known to delay ocular elongation have also been shown to lead to transient thickening of the choroid in animal research. Myopic defocus (40, 41), pharmacological agents such as muscarinic antagonists (42), and dopamine agonists (43), as well as environmental factors such as increased light exposure (44), have all been shown to lead to transient increases in CT.

Our study has several limitations. The relatively small number of eyes with different MMD severity grades limited us to further explore the changes of CT and CPA after PSR surgery in each META-PM category group. A controlled prospective study with larger sample size was needed to further study the role of PSR in pathologic myopic choroidal flow changes with different MMD severity grades. The follow-up time is relatively short in our study. However, according to our studies, the CT and CPA were increased shortly after PSR and tended to return to preoperative status at 1 months' follow-up time. It can be inferred that the longer the follow-up time, the more difficult to detect the changes of CT and choroidal blood flow.

In conclusion, we demonstrated that CT and CPA were independently associated with MMD. The CT and choroidal blood flow increased significantly in patients with high myopia who underwent PSR surgery in a short period of time. However, whether this transient improvement of the choroidal circulation could prevent the development of high myopia is still unclear and requires further long-term investigation with larger samples.

## Data Availability Statement

The raw data supporting the conclusions of this article will be made available by the authors, without undue reservation.

## Ethics Statement

The studies involving human participants were reviewed and approved by institutional ethic committee of Beijing Tongren Hospital, Capital Medical University. The patients/participants provided their written informed consent to participate in this study.

## Author Contributions

ZZ, YQ, WWe, and WL designed this study. ZZ and YQ collected and measured data. ZZ, YQ, Z-BJ, and WWa analyzed data. ZZ and YQ wrote this article. Z-BJ and AD revised the manuscript. All authors discussed the results and commented on the manuscript.

## Conflict of Interest

The authors declare that the research was conducted in the absence of any commercial or financial relationships that could be construed as a potential conflict of interest.
